# AMOTL1 enhances YAP1 stability and promotes YAP1-driven gastric oncogenesis

**DOI:** 10.1038/s41388-020-1293-5

**Published:** 2020-04-20

**Authors:** Yuhang Zhou, Jinglin Zhang, Hui Li, Tingting Huang, Chi Chun Wong, Feng Wu, Man Wu, Nuoqing Weng, Liping Liu, Alfred S. L. Cheng, Jun Yu, Nathalie Wong, Kwok Wai Lo, Patrick M. K. Tang, Wei Kang, Ka Fai To

**Affiliations:** 1 Department of Anatomical and Cellular Pathology, State Key Laboratory of Translational Oncology, Prince of Wales Hospital, The Chinese University of Hong Kong, Hong Kong, SAR PR China; 2 Institute of Digestive Disease, State Key Laboratory of Digestive Disease, The Chinese University of Hong Kong, Hong Kong, SAR PR China; 3 Li Ka Shing Institute of Health Science, Sir Y.K. Pao Cancer Center, The Chinese University of Hong Kong, Hong Kong, SAR PR China; 4 Sun Yat-Sen University Cancer Center, Guangzhou, Guangdong Province China; 5 Department of Hepatobiliary and Pancreatic Surgery, Shenzhen People’s Hospital, Second Clinical Medical College of Jinan University, Shenzhen, Guangdong Province PR China; 6 School of Biomedical Sciences, The Chinese University of Hong Kong, Hong Kong, SAR PR China; 7 Department of Medicine and Therapeutics, The Chinese University of Hong Kong, Hong Kong, SAR PR China

**Keywords:** Gastric cancer, Tumour biomarkers, Protein transport

## Abstract

Hippo signaling functions to limit cellular growth, but the aberrant nuclear accumulation of its downstream YAP1 leads to carcinogenesis. YAP1/TEAD complex activates the oncogenic downstream transcription, such as CTGF and c-Myc. How YAP1 is protected in the cytoplasm from ubiquitin-mediated degradation remains elusive. In this study, a member of Angiomotin (Motin) family, AMOTL1 (Angiomotin Like 1), was screened out as the only one to promote YAP1 nuclear accumulation by several clinical cohorts, which was further confirmed by the cellular functional assays. The interaction between YAP1 and AMOTL1 was suggested by co-immunoprecipitation and immunofluorescent staining. The clinical significance of the AMOTL1–YAP1–CTGF axis in gastric cancer (GC) was analyzed by multiple clinical cohorts. Moreover, the therapeutic effect of targeting the oncogenic axis was appraised by drug-sensitivity tests and xenograft-formation assays. The upregulation of AMOTL1 is associated with unfavorable clinical outcomes of GC, and knocking down AMOTL1 impairs its oncogenic properties. The cytoplasmic interaction between AMOTL1 and YAP1 protects each other from ubiquitin-mediated degradation. AMOTL1 promotes YAP1 translocation into the nuclei to activate the downstream expression, such as CTGF. Knocking down AMOTL1, YAP1, and CTGF enhances the therapeutic efficacies of the first-line anticancer drugs. Taken together, AMOTL1 plays an oncogenic role in gastric carcinogenesis through interacting with YAP1 and promoting its nuclear accumulation. A combination of AMOTL1, YAP1, and CTGF expression might serve as a surrogate of Hippo activation status. The co-activation of the AMOTL1/YAP1–CTGF axis is associated with poor clinical outcomes of GC patients, and targeting this oncogenic axis may enhance the chemotherapeutic effects.

## Introduction

Gastric cancer (GC) ranks as the fourth common malignancy globally [1]. It is a heterogeneous disease with multiple environmental risk factors [2]. Given the late diagnosis, the 5-year survival rate of the patients who are suffering from GC is up to 30%. Studies have indicated that multiple signaling pathways promote gastric oncogenesis [3–5]. Recent findings have revealed the emerging role of Hippo signaling in carcinogenesis [5–7], whose dysregulation drives tumor initiation and progression [8, 9]. Yes-associated protein 1 (YAP1), the downstream of the Hippo pathway, has been identified to promote tumorigenesis in different kinds of tumor types. Previously, our group has identified the cancer-driving role of YAP1 in GC through its nuclear accumulation [10]. Targeting YAP1 by small molecules might serve as an intervention strategy for GC patients [11, 12].

As a transcription co-activator, YAP1 cooperates with transcriptional factors to bind with targeted DNA regions, thus transducing the proliferative signals through activating the downstream transcription [8]. TEAD family, mainly TEAD1/4, has been proved as the predominant transcriptional factor of YAP1 in gastric tumorigenesis [13]. Under normal circumstances, the upstreams of the Hippo pathway, MST1/2 and LATS1/2, sequester YAP1 and promote YAP1 degradation in the cytoplasm. However, during gastric carcinogenesis, YAP1 is overexpressed in the cytoplasm. How YAP1 manages to be translocated into the nucleus and avoids to be degraded in the cytoplasm has not been well elucidated in GC.

Among the interactants of YAP1, Angiomotin (Motin or AMOT) family has been reported for its functions during tumorigenesis [14]. This family consists of three members in mammalian cells: AMOT, AMOT-Like 1 (AMOTL1), and AMOT-Like 2 (AMOTL2). They all possess the PPXY motifs, which endow the members with the ability to bind with the WW domains of YAP1 [14]. Nevertheless, the behavior of Motin–YAP1 interaction displays controversial consequences [14]. In GC, their roles have not been explored. Therefore, the current study aims to identify the role of the Motin and its involvement in the Hippo pathway in gastric oncogenesis.

## Results

### Abundant AMOTL1 in GC indicates poor clinical outcomes

**Fig. 1:**
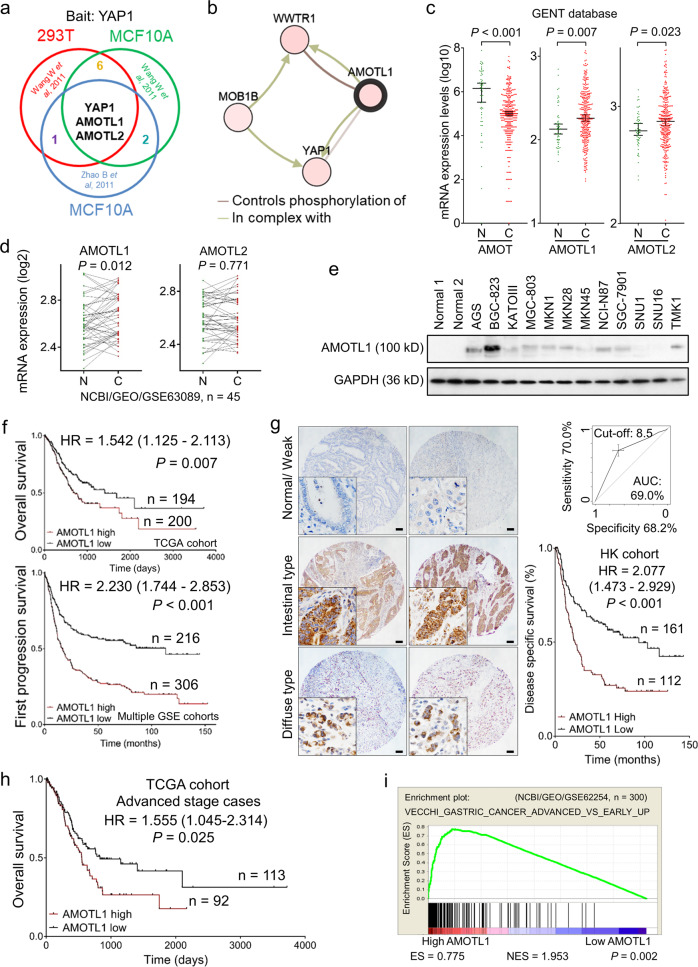
AMOTL1 is overexpressed and correlated with poor survival in GC. **a** Multiple published mass spectrometry results indicated that AMOTL1 and AMOTL2 are the common binding partners of YAP1. **b** Based on the TCGA database, the network analysis revealed the putative interactive capability between AMOTL1 and YAP1. **c** Overall primary cases (GENT database, *n* = 368) suggested that AMOT (*P* < 0.001) is decreased in normal tissues, while AMOTL1 (*P* = 0.007) and AMOTL2 (*P* = 0.023) are upregulated in GC. **d** Paired samples (NCBI/GEO/GSE63089) indicated that AMOTL1 (*P* = 0.012) is abundant in GC, yet the alteration of AMOTL2 is not statistically significant. **e** In total, 7 out of 12 GC cell lines possess overexpression of AMOTL1, compared with the other two normal gastric epithelial cell lines. **f** High AMOTL1 expression predicts poor survival in GC patients (overall cases, *P* = 0.007, *n* = 394, TCGA cohort; first progression survival, *P* < 0.001, *n* = 522, multiple GSE cohort). **g** The figures of IHC represent score 0 (Normal/Weak), score 12 (Intestinal type), and score 12 (Diffuse type), respectively (scale bars are 200 μm). A cut-off value of 8.5 was set accordingly (upper right), and AMOTL1 upregulation is associated with worse outcomes (disease-specific survival, *P* < 0.001, *n* = 273, HK cohort) (lower right). **h** The hazard of overexpressed AMOTL1 is more obvious among GC patients in the advanced stage, compared with that in the early stage (overall cases, *P* = 0.025, *n* = 205, TCGA cohort). **i** GSEA demonstrates a positive correlation between AMOTL1 enrichment and advanced stage of GC (*P* = 0.002). ES enrichment score; NES normalized enrichment score.

To explore the interactive components of YAP1, mass spectrometry results from other published reports have been analyzed [15, 16]. AMOTL1 and AMOTL2 are confirmed as the most common binding partners for YAP1 (Fig. 1a). Furthermore, AMOTL1 was predicted to interact with the WW domain of YAP1 (cBioportal) (Fig. 1b). Given the contradictory roles of the family members in oncogenesis [14], their expression pattern was examined in primary gastric samples (GENT database) and gastric cell lines. Apparently, AMOT is downregulated in GC (*n* = 368, *P* < 0.001), while AMOTL1 (*n* = 368, *P* = 0.007) and AMOTL2 (*n* = 368, *P* = 0.023) are abundantly expressed (Fig. 1c). Meanwhile, the paired samples in another Chinese cohort (*n* = 45, NCBI/GEO/GSE63089) suggested that AMOTL1 (*P* = 0.012) is enriched in tumor samples, but the alteration of AMOTL2 in paired GC (*P* = 0.771) shows no difference (Fig. 1d). In GC cell lines, AMOT was barely expressed, but the levels of AMOTL1 and AMOTL2 were relatively high (Supplementary File: Fig. S1a). From the protein level, AMOTL1 exhibited abundance in 7 out of 12 GC cell lines compared with normal controls (Fig. 1e). Its upregulation might be positively regulated by putative transcriptional factors, STAT5A (*r* = 0.264, *P* < 0.001, *n* = 415) and PBX1 (*r* = 0.597, *P* < 0.001, *n* = 415) (Supplementary File: Fig. S1b). In primary samples (TCGA and KM plotter cohorts), AMOT and AMOTL2 expressions are insufficient to indicate prognosis (*n* = 397, *P* > 0.05; Supplementary file: Fig. S1c, d). However, high expression of AMOTL1 predicts poor survival in GC patients (*n* = 394, *P* = 0.007, TCGA cohort; *n* = 522, *P* < 0.001, multiple GSE cohorts, Fig. 1f). AMOTL1 was predominantly localized in the cytoplasm, which was detected by immunohistochemistry in tissue microarray. Its high expression correlates with unfavorable outcomes (*n* = 273, *P* < 0.001, HK cohort, Fig. 1g). In GC cases, the expression level of AMOTL1 is highly correlated with the advanced stages (*n* = 205, *P* = 0.025, TCGA cohort, Supplementary file: Fig. S1e; Supplementary file: Tables S1, S2) and is also associated with poor overall survival (*n* = 205, *P* = 0.025, TCGA cohort, Fig. 1h). In addition, enrichment of AMOTL1 is found in advanced-stage GC cases (*P* = 0.002, NCBI/GEO/GSE62254, Fig. 1i) instead of the early-stage GC (*n* = 175, *P* = 0.054, TCGA cohort, Supplementary file: Fig. S1f). Given the concordant findings of AMOTL1 in multiple primary GC cohorts, its functional role and molecular mechanisms were further investigated.

### AMOTL1 knockdown (KD) retards oncogenic features of GC cell lines

**Fig. 2:**
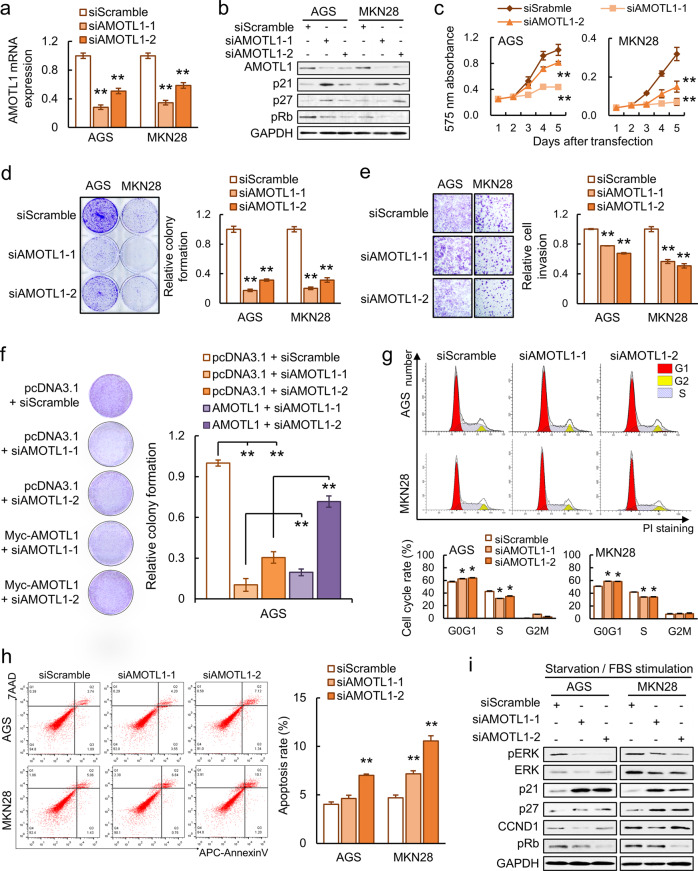
Knocking down AMOTL1 exerts anti-tumor effects. **a** The KD efficiency of siAMOTL1 was confirmed by qRT-PCR. **b** AMOTL1 KD, p21, and p27 were upregulated, whereas pRb was reduced. **c** MTT assay suggested that siAMOTL1 suppressed GC cellular growth (**P < 0.001). **d** Monolayer colony formation assay indicated a slower proliferation of GC cells after AMOTL1 KD (**P < 0.001). **e** Cellular invasive ability was retarded by AMOTL1 deactivation (**P < 0.001). **f** Re-expression of AMOTL1 partly restored the KD effect of siRNAs on AGS. **g** siAMOTL1 resulted in G1-phase arrest in GC cell lines (*P < 0.05). **h** AMOTL1 KD increased cellular apoptosis (**P < 0.001). **i** During the starvation/FBS stimulation, GC cells with siAMOTL1 exhibited lower levels of ERK, pERK, CCND1, and pRb, while the expressions of p21 and p27 were elevated.

Given the abundance of AMOTL1 in GC, small-interfering RNA (siRNA)-mediated KD was applied to analyze its function in vitro. Based on a prominent AMOTL1 KD efficiency from both mRNA (**P < 0.001, Fig. 2a) and protein levels (Fig. 2b) in AGS and MKN28 cells, cellular proliferative rate (**P < 0.001; MTT proliferation assays, Fig. 2c), monolayer colony formation assay (**P < 0.001, Fig. 2d), and cell-invasive abilities (**P < 0.001, Fig. 2e) were all significantly inhibited. A third GC cell line, BGC-823, was also applied to verify the suppressive effect of siAMOTL1 (Supplementary File: Fig. S2a, b, upper panel). Rescue experiments were performed to confirm the KD veracity of siAMOTL1s (**P < 0.001, Fig. 2f). To investigate the mechanism of growth suppression, cell-cycle distribution after siAMOTL1 transfection was examined. A high percentage of G0/G1-phase cells was detected in siAMOTL1 transfectants (*P < 0.05, Fig. 2g). Moreover, G0/G1-phase cell-cycle arrest was confirmed by the upregulation of p21/p27 and the decrease in pRb by Western blot analysis (Fig. 2b). Apart from cell-cycle arrest, the results from 7AAD and Annexin V double staining indicated that AMOTL1 KD also enhanced both late- and early-stage apoptosis (**P < 0.001, Fig. 2h). Regarding the oncogenic function of AMOTL1 in GC cells, mechanistic studies were conducted to identify the underlying signaling pathway. The stimulation assays were performed. The cells were deprived of serum from 24 h to achieve synchronization, followed by 10% FBS medium stimulation for 15 min. pERK1/2 was decreased in cells with siAMOTL1 transfectants compared with siScramble controls (Fig. 2i), indicating that AMOTL1 KD weakened the MAPK-mediated signal transduction for proliferation.

### AMOTL1 co-localizes with YAP1 in the cytoplasm and promotes YAP1 nuclear translocation

**Fig. 3:**
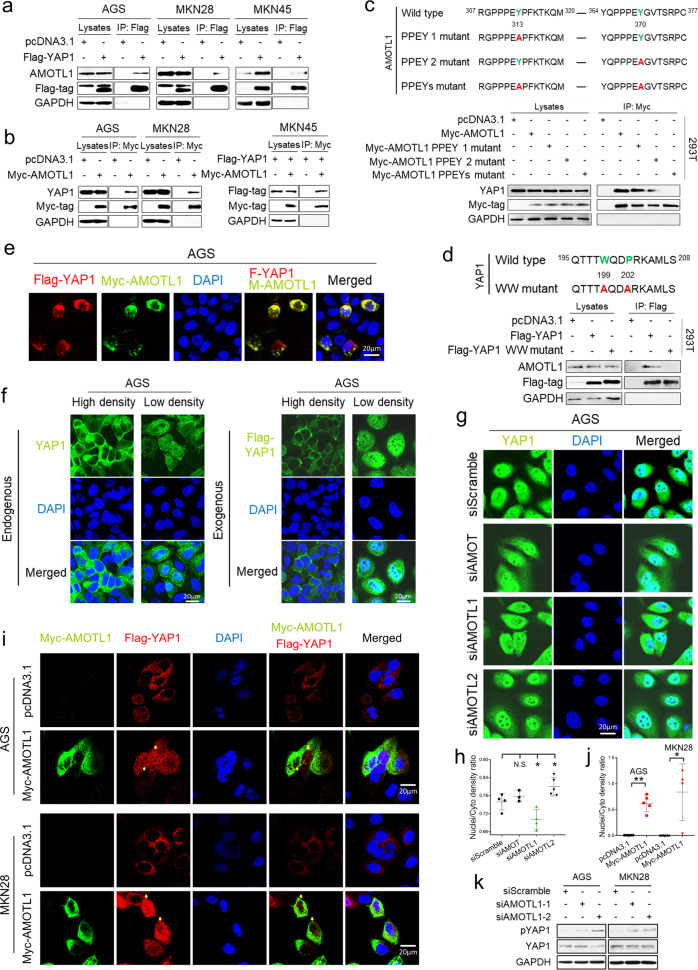
AMOTL1 binds with YAP1 in GC and promotes YAP1 nuclear accumulation. **a** Through co-IP assay, AMOTL1 binds with YAP1 in multiple GC cell lines. **b** Both endogenous and exogenous YAP1 also interact with AMOTL1 in GC cells. **c** Point mutations were designed based on the protein structures of AMOTL1 (PPEY domains) and **d** YAP1 (WW domain). Mutants failed to interact with each other physically. **e** Immunocytochemistry staining indicated a co-localization of YAP1 and AMOTL1 proteins in the cytoplasm among low-density cells. **f** With or without exogenous YAP1 protein, the Hippo pathway is activated when cells are in a high density, which was illustrated by the cytoplasmic retention of YAP1. **g**, **h** A weaker nuclear signal of YAP1 was detected in the siAMOTL1 group compared with siAMOT and siAMOTL2 groups, which were quantified by fluorescent-density ratio of nuclei versus cytoplasm (Nuclei/Cyto) (N.S. not significant; *P < 0.01). **i**, **j** In GC cells, overexpression of AMOTL1 raised nuclear YAP1 accumulation. **k** AMOTL1 knockdown increased YAP1 phosphorylation.

The interaction between AMOTL1 and YAP1 was validated by co-immunoprecipitation (co-IP) in GC cell lines (Fig. 3a, b). MKN45, a cell line with YAP1 homozygous deletion, was also applied to investigate the AMOTL1–YAP1 interplay. Furthermore, the mutants of YAP1 (WW domain, YAP1^W199A/P202A^, Addgene #17792) and AMOTL1 (PPEY domains, AMOTL1^Y313A^, AMOTL1^Y370A^, and AMOTL1^Y313A/Y370A^) were applied to confirm if they still interact as previously reported [14]. As a consequence, the mutation of either both PPEY domains in AMOTL1 or the WW domain in YAP1 abolished the interaction between these two proteins (Fig. 3c, d). For a more detailed investigation of the interaction, immunocytochemistry staining was performed to detect the protein localization. As indicated by Fig. 3e, YAP1 and AMOTL1 were co-localized in the cytoplasm. As YAP1 lies in the center of the Hippo cascade, we checked the localization of YAP1 in a high or low cell-density condition. Under high cellular density, both endogenous and exogenous YAP1 was predominantly retained in the cytoplasm, while low cellular density allowed YAP1 to translocate to the nucleus (Fig. 3f). In addition, only siAMOTL1, instead of siAMOT and siAMOTL2, inhibited YAP1 cytoplasm-to-nuclear translocation (Fig. 3g; N.S., not significant; *P < 0.01, Fig. 3h). To take a step further, siAMOTL1 decreased the nuclear entrance for both endogenous and exogenous YAP1 (Supplementary File: Fig. S2c, d), which were quantified by fluorescent-density ratio of nuclei versus cytoplasm. On the contrary, overexpression of AMOTL1 increased YAP1 nuclear aggregation (Fig. 3i; *P < 0.01; **P < 0.001, Fig. 3j). There are two states of YAP1 in cells: functional YAP1 and nonfunctional YAP1 (phosphorylated YAP1, pYAP1) [17]. We extracted the proteins in the cytoplasm after siAMOTL1 transfection, and found that AMOTL1 KD activates pYAP1 (Fig. 3k). Given the evidence, we proposed that AMOTL1 might have a protective effect on YAP1 from degradation, and therefore allows the nuclear translocation of YAP1.

### AMOTL1 prevents YAP1 from ubiquitin-mediated degradation

**Fig. 4:**
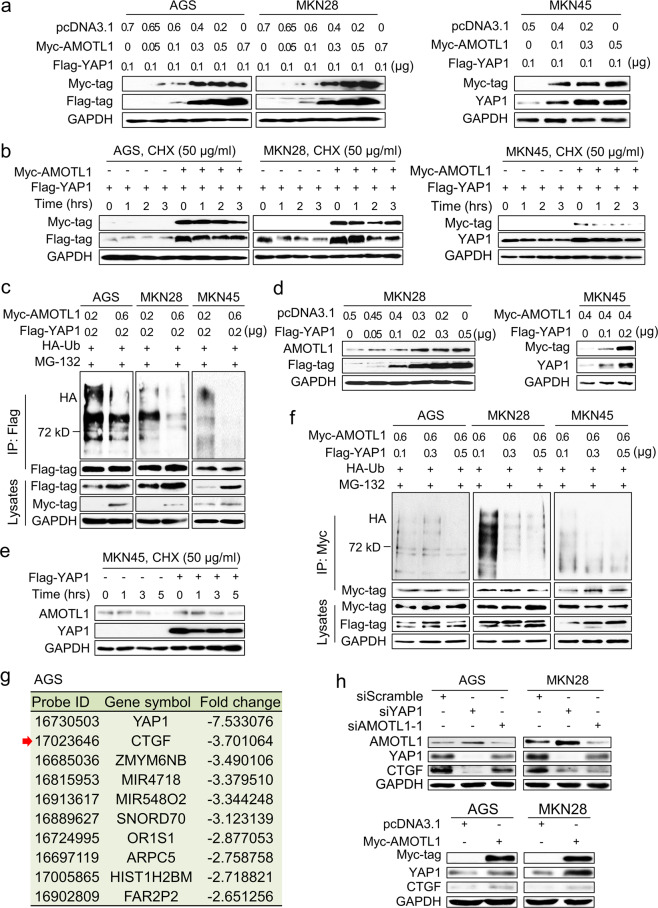
The interaction between AMOTL1 and YAP1 protects each other from degradation. **a** AMOTL1 prevented YAP1 from degradation in a dosage-dependent manner. **b** The half-life of YAP1 protein was elongated by AMOTL1 overexpression. **c** YAP1 degradation is mainly through ubiquitination, which was reduced by AMOTL1 overexpression in a dosage-dependent manner. **d** YAP1 also protected AMOTL1 from degradation dosage-dependently. **e** YAP1 prolonged the half-life of AMOTL1 protein. **f** YAP1 prevented AMOTL1 ubiquitination in a dosage-dependent manner. **g** The list of top ten genes that were downregulated in YAP1-depleted cells. CTGF was the top one. **h** Western blot analysis confirmed that both siAMOTL1 and siYAP1 suppressed CTGF expression, while AMOTL1 overexpression increased YAP1 and CTGF protein levels.

In GC cell lines AGS, MKN28, and MKN45, with growing dosages of the AMOTL1 transfection, the YAP1 protein amount was increased according to AMOTL1 (Fig. 4a). Further, we tried to elucidate whether AMOTL1 regulates the expression level of YAP1. Cyclohexamide (CHX), an inhibitor of protein biosynthesis, leads to protein degradation along with time. CHX was used to induce protein degradation in GC cells. In the absence of ectopic AMOTL1, YAP1 was degraded with time. However, in the existence of AMOTL1, the total amount of YAP1 was preserved, and the half-life of the YAP1 protein was extended (Fig. 4b). Next, we would like to elucidate how AMOTL1 prevents YAP1 from degradation. There are two main pathways of protein degradation: Ubiquitin/Proteasome System (UPS) is a major one, and Lysosomal Proteolysis is a minor one. To confirm whether UPS governs the YAP1 degradation, we transfected HA-tagged ubiquitin (HA-Ub) into GC cell lines to monitor UPS, and added MG132 to inhibit proteasome. With the increasing exogenous AMOTL1, the ubiquitination-mediated YAP1 degradation was significantly eliminated. It indicated that AMOTL1 may contribute to keep YAP1 from degradation (Fig. 4c). On the other hand, with an increasing amount of exogenous YAP1, AMOTL1 proteins were also remained in a dose-dependent manner (Fig. 4d). Under the treatment of CHX, endogenous YAP1 was dismissed and AMOTL1 was also decreased along with the treatment time. However, exogenous YAP1 attenuated the degradation of AMOTL1 in GC cells (Fig. 4e). An increasing amount of YAP1 was also found preventing AMOTL1 from ubiquitin-mediated degradation (Fig. 4f). In addition, the ubiquitination of YAP1 was much stronger than that of AMOTL1 (Supplementary File: Fig. S2e), suggesting a more crucial protection role of AMOTL1 in YAP1-driven GC. Through expression profiling, connective tissue growth factor (CTGF) was confirmed to be the main downstream target of YAP1 in GC (Fig. 4g and Supplementary file: Table S3). YAP1 KD decreased its downstream target CTGF, while AMOTL1 KD caused the reduction of YAP1 as well as CTGF in GC cell lines; meanwhile, overexpression of AMOTL1 upregulated both YAP1 and CTGF (Fig. 4h and Supplementary File: Fig. S2b, lower panel). Taken together, our data indicated that interaction between AMOTL1 and YAP1 stabilizes each other in the cytoplasm, and the stabilization ensures YAP1 nuclear accumulation and transcriptional potential.

### Co-overexpression of YAP1–AMOTL1–CTGF indicates poor clinical outcomes

**Fig. 5:**
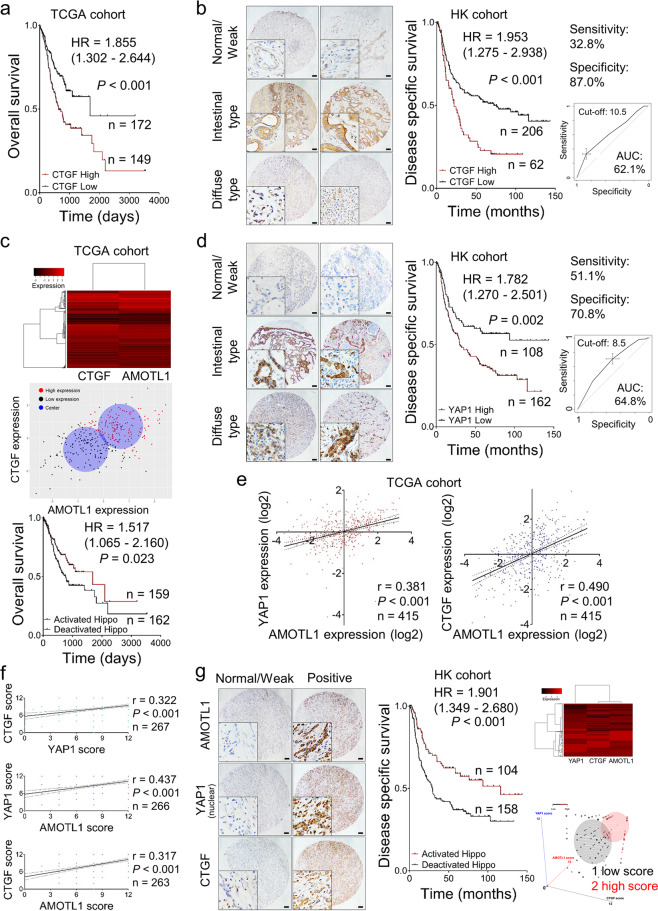
The activation of AMOTL1-nuclear YAP1–CTGF axis is associated with poor clinical outcomes. **a** High expression of CTGF indicated poor survival in the TCGA cohort (overall cases, *P* < 0.001, *n* = 321). **b** In the HK cohort, the abundant CTGF also predicted poor survival (middle) (disease-specific, *P* < 0.001, *n* = 268). The cut-off value was identified according to ROC (right). **c** Unsupervised clustering of the patients in the TCGA cohort regarding both AMOTL1 and CTGF expression (upper) was presented in a two-dimensional coordinate (middle). The survival curve after grouping was shown (lower) (overall cases, *P* = 0.023, *n* = 321, TCGA cohort). **d** YAP1 nuclear aggregation (left) indicates worse outcomes for the patients (middle) (disease-specific survival, *P* = 0.002, *n* = 268). The grouping was based on the ROC (right). **e** In the TCGA dataset, AMOTL1 expression is positively correlated with YAP1 (left) (*r* = 0.381, *P* < 0.001, *n* = 415) and CTGF (right) (*r* = 0.490, *P* < 0.001, *n* = 415). **f** In the HK cohort, a pairwise positive association was detected within AMOTL1, nuclear YAP1, and CTGF. **g** Based on the intensity of IHC signals, co-expression of AMOTL1, YAP1 (nuclear), and CTGF was stratified into two groups: Normal/Weak group and Positive group, referring to “activated Hippo” and “deactivated Hippo”, respectively. Identical cases were demonstrated in the left panel, and clustering analysis was shown in the right panel. Further, deactivated Hippo group is related to worse prognosis of GC patients (middle panel) (disease-specific survival, *P* < 0.001, *n* = 268).

Based on a TCGA cohort, the overabundance of CTGF mRNA was associated with worse outcomes (*n* = 321, *P* < 0.001, Fig. 5a). Our Hong Kong cohort also showed a similar result from the protein level (*n* = 268, *P* < 0.001, Fig. 5b). CTGF was predominantly expressed in the cytoplasm both in intestinal and diffuse types of GC. Besides, the clinical information of the TCGA cohort suggested that both AMOTL1 (*P* = 0.016) and CTGF (*P* = 0.008) are related to worst-survival cases, respectively (Cox regression analysis, Supplementary File: Table S4). Given their prognostic potential, we combined these two biomarkers through unsupervised clustering to check whether the combination could distinguish the survival situation. However, the combination did not present a better differentiation (*n* = 321, *P* = 0.023, Fig. 5c). The less power of differentiation might be caused by the exclusion of YAP1. As the downstream effector of Hippo, YAP1 links AMOTL1 and CTGF during gastric carcinogenesis. Our previous findings demonstrated the importance of nuclear accumulation of YAP1 in GC [10]. In the current study, we enlarged the sample size and reconfirmed the previous conclusion that YAP1 overexpression correlates with poor survival (*n* = 270, *P* = 0.002, Fig. 5d). The clinical relevance of both AMOTL1 and CTGF protein expression (*P* < 0.001) was verified as well (Cox regression analysis, Supplementary File: Table S5). Moreover, the positive correlations between the expression of any two factors among AMOTL1, YAP1, and CTGF were observed both in TCGA and HK cohorts, respectively (Fig. 5e, f). In addition, we observed a significant decrease (*r* = −0.958, *P* = 0.042) of the difference between AMOTL1 and nuclear YAP1, which meant that they are co-expressed mainly in the advanced-stage GCs (Supplementary File: Fig. S2f). Perhaps, this might explain why AMOTL1 has a significant role in advanced GC cases (Fig. 1h) instead of in the early stage (Supplementary Fig. S1f). After clustering these three markers, the cases with AMOTL1, CTGF overexpression, and YAP1 nuclear accumulation, named “deactivated Hippo” group, indicated unfavorable clinical outcomes (Fig. 5g).

### Verteporfin quenches AMOTL1–YAP1 and represses gastric oncogenesis

**Fig. 6:**
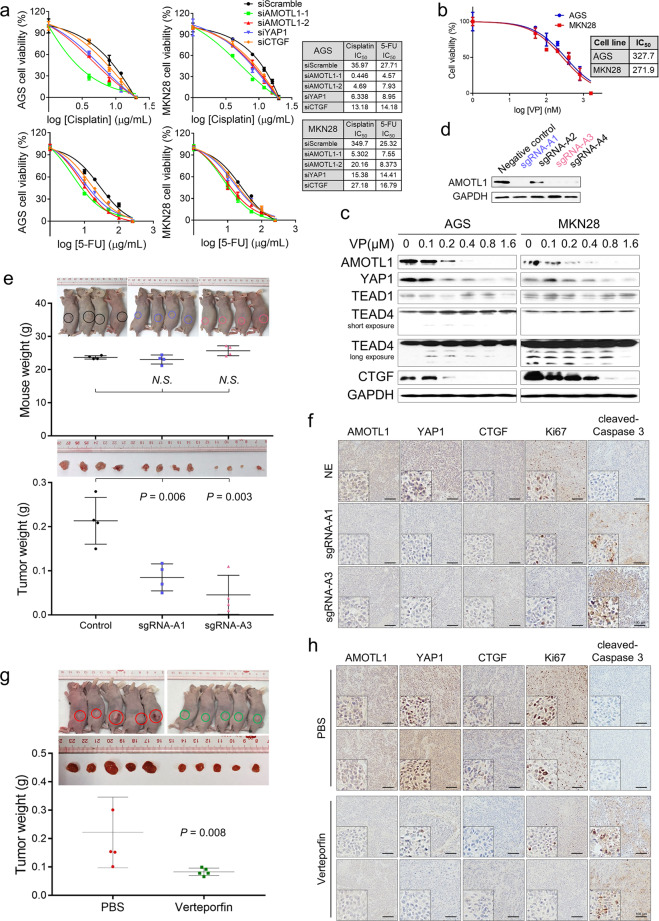
Targeting AMOTL1–YAP1 enhances first-line chemotherapeutic efficacies. **a** The efficacies of the first-line anti-cancer drugs (Cisplatin and 5-FU) were assessed on GC cells after knocking down AMOTL1, YAP1, CTGF, and control, respectively. **b** The IC_50_ of VP was evaluated on GC cell lines. **c** VP promotes AMOTL1 and YAP1 degradation in a dosage-dependent manner. **d** CRISPR/Cas9 mediated AMOTL1 KO by targeting multiple genetic sites. **e** Xenograft images of AMOTL1-KO and the negative control groups. Weights of the mice and the tumors were measured accordingly (N.S., not significant). **f** Representative IHC images of AMOTL1, YAP1, CTGF, Ki67, and cleaved-Caspase 3 on the xenograft samples from different groups. **g** VP administration decreased xenograft formation (*P* = 0.008). **h** IHC staining images of AMOTL1, YAP1, CTGF, Ki67, and cleaved-Caspase 3 on the xenografts in PBS and VP treatment groups.

Besides the prognostic capability, there is also a therapeutic potential that lies behind this AMOTL1/YAP1–CTGF axis. AMOTL1, YAP1, and CTGF were silenced, respectively, in GC cell lines followed with the treatment of first-line anti-cancer drugs (Cisplatin and 5-FU). Notably, silencing AMOTL1 resulted in a remarkable enhancement of the sensitivity of GC cells to chemodrugs, which was characterized by the reduced IC_50_ values (Fig. 6a). This observation hinted that deactivating AMOTL1 might be the most efficient way of targeting this axis, and it could be accompanied by anti-cancer chemotherapy, especially for cisplatin treatment. On the other hand, Verteporfin (VP) has been widely reported to degrade YAP1 [9, 13]. After calculation of the IC_50_ value of VP on two GC cell lines (Fig. 6b), we performed Western blot analysis with different concentrations of VP. The data demonstrated that increased concentration of VP also caused deterioration of AMOTL1 and CTGF, while it had no obvious effect on the expression of TEADs (Fig. 6c). We further established CRISPR/Cas9-based stable BGC-823 cell lines with AMOTL1 KO. Two sgRNAs, sgRNA-A1 and sgRNA-A3, were selected for tumor formation assays (Fig. 6d). In the xenograft experiments, AMOTL1-KO-derived tumors were significantly smaller and lighter in weights compared with the negative control ones (Fig. 6e). By immunohistochemistry, the expression of AMOTL1, YAP1, CTGF, and Ki67 was uniformly downregulated in KO groups, while the apoptotic marker cleaved-Caspase 3 was activated (Fig. 6f). Moreover, VP treatment was able to diminish xenograft formation significantly (Fig. 6g). Apart from the inhibition on AMOTL1, YAP1, and CTGF, VP suppressed cell growth indicated by the downregulation of Ki67 and promoted cell apoptosis in vivo (Fig. 6h).

**Fig. 7:**
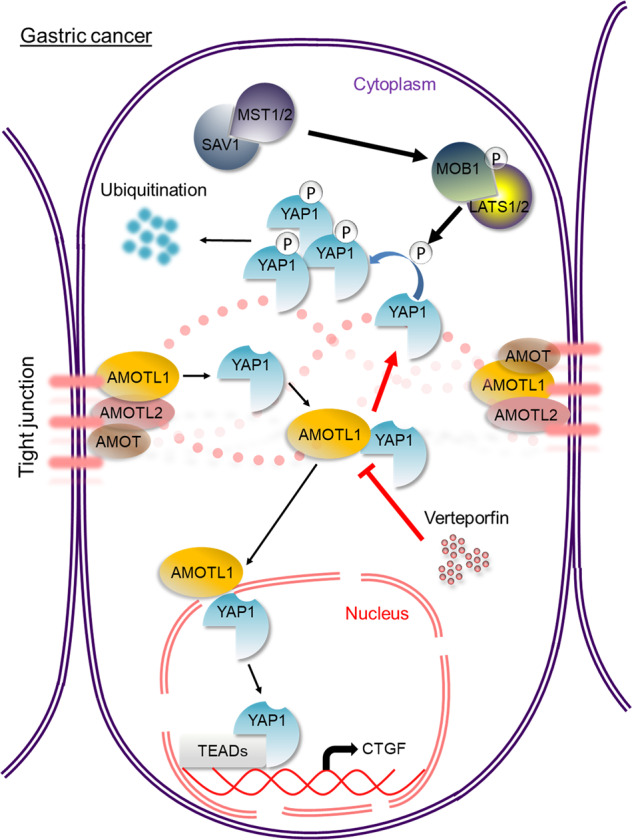
Overall schematic presentation. When Hippo is activated, it promotes YAP1 to degrade ubiquitination in the cytoplasm. In GC cells, abundant AMOTL1 protects YAP1, reduces its phosphorylation, and promotes its nuclear translocation. In the nucleus, YAP1 binds with transcriptional factor TEAD to activate the downstream CTGF expression and thus to drive gastric carcinogenesis. The administration of small-molecule VP blocks the interaction of AMOTL1 and YAP1 and quenches their oncogenic properties.

Combining the above results, we provided the oncogenic AMOTL1/YAP1–CTGF axis in driving gastric tumorigenesis. In normal gastric epithelium cells with activated Hippo pathway, abundant YAP1 will be phosphorylated, resulting in its cytoplasmic retention and ubiquitination-mediated degradation. However, in GC cells, AMOTL1 is upregulated and binds to YAP1 to prevent its degradation. This successfully raises YAP1 nuclear accumulation. Consequently, through the transcriptional factor TEAD, YAP1 promotes CTGF expression during gastric tumorigenesis. VP, a small molecule quenching AMOTL1–YAP1 complex, can be employed to target AMOTL1–YAP1 and suppress tumor growth (Fig. 7).

## Discussion

Hippo–YAP1 signaling is among the most prominent intracellular pathways in gastric carcinogenesis. Hippo functions as a tumor suppressor in GC, which is concordant with other cancer types [18–20]. Our previous works have delineated the role of YAP1 in GC and its regulation from the post-translational level [11, 12]. Due to the dysfunction of the Hippo pathway upstream and the regulators, the enrichment of nuclear YAP1 drives gastric oncogenesis. Following the nuclear existence of YAP1, we validated the necessity of TEAD family, particularly TEAD1/4, as YAP1-binding partner and transcriptional factor in GC [13]. Through their interactions, YAP1 transduces the proliferative or oncogenic signals to the downstream, such as CTGF, Cyr61, and c-Myc [8]. The other group results also supported the conclusions as well [21–23]. However, YAP1 could still be localized in the cytoplasm for degradation.

The Motin family members, AMOT, AMOTL1, and AMOTL2, have been reported to bind with YAP1 in the cytoplasm. The Motin family has been revealed to participate in angiogenesis [24–26] and other physiological processes, such as embryonic development [27, 28]. During oncogenesis, studies have also pointed out that the Motin family binds with YAP1, but the functional role is controversial. The consequences of Motin–YAP1 interplays vary from context to context [14]. Some reports proposed Motins as cancer promoters [29, 30], while some believed that Motins control tumor growth by mediating YAP1 [16, 31, 32]. The functions of Motins in gastric carcinogenesis are little known. The only related study was focusing on AMOT, in which the authors presented a positive correlation between decreased AMOT and abundant YAP1 in GC patients with worse outcomes [33]. We found that only AMOTL1 showed a concordant overexpression in multiple GC cohorts. In the three members, only AMOTL1 plays an oncogenic role by promoting YAP1 nuclear translocation. After identifying the oncogenic property of AMOTL1 in GC, we revealed the detailed mechanisms of AMOTL1 in the Hippo pathway. AMOTL1 acts as an oncoprotein by protecting YAP1 from degradation in the cytoplasm. Furthermore, our data demonstrated that the phosphorylated YAP1 (pYAP1) was increased when AMOTL1 was knocked down, suggesting that without the presence of AMOTL1, YAP1 tended to be degraded.

It has been well established that CTGF serves as a prognostic biomarker for GC patients, especially in cases of the advanced stage [12, 34]. Although CTGF has been involved in other cascades [35], it is regulated predominantly by Hippo signaling [8, 12]. As a direct downstream of YAP1, CTGF expression is dramatically activated after YAP1 nuclear location. The prognostic value of YAP1 was reported in our previous work [10]. In our current cohort, a larger sample size was achieved to reconfirm our previous conclusion. Given the central role of YAP1, we chose three representatives in this process (cytoplasmic stage, nuclear accumulation, and transcription effect) to stand for the activation status of Hippo pathway. The high binding affinity and the protective effect toward YAP1 allow AMOTL1 to represent for the cytoplasmic stage of YAP1. Meanwhile, CTGF is a predominant downstream effector of YAP1 nuclear translocation, and its overexpression represents the activation of YAP1. Through unsupervised clustering, the cases with simultaneous enrichments of AMOTL1, nuclear YAP1, and CTGF were represented as “Hippo-deactivated”, which were characterized by the worse prognosis. In other words, activation of this axis (AMOTL1-nuclear YAP1–CTGF) indicates the Hippo’s deactivation states. In fact, another study has proposed the concept of Hippo “on” or “off” in an earlier time. However, the study only obscurely referred to YAP1 “nuclear accumulation” or “cytoplasmic retention” [36]. Based on our current results, we employed the AMOTL1-nuclear YAP1–CTGF axis to specify the concept of Hippo activation and deactivation.

Apart from the prognostic indicator, there is a more important therapeutic potential that lies in the AMOTL1-nuclear YAP1–CTGF axis. Knocking down this oncogenic cascade enhances the efficacies of the first-line anti-cancer drugs (Cisplatin and 5-FU). Meanwhile, VP was reported to be one of the small molecules that specifically target this axis. In fact, VP has been wildly proposed to downregulate YAP1 in various types of cancers [18, 37–39], especially the capability of VP that disrupts the interaction between YAP1 and transcriptional partners. However, a detailed mechanism still remains to be explored. In our current study, we found that VP suppressed multiple targets, including AMOTL1, YAP1, and CTGF, which might serve as a desirable option to help GC patients to achieve satisfied therapeutic effects during chemotherapy.

## Conclusion

In the current study, we proposed the oncogenic AMOTL1-nuclear YAP1–CTGF axis in gastric carcinogenesis. It not only serves as a prognostic indicator for GC patients, but also provides a rational therapeutic target in this personalized medicine era.

## Materials and methods

### Cell lines and clinical samples

Sources and culture methods of the 12 human GC cell lines (AGS, BGC-823, KATOIII, MGC-803, MKN1, MNK28, MKN45, NCI-N87, SGC-7901, SNU1, SNU16, and TMK1) and two normal gastric epithelial cell lines were described before [40]. All 278 selected patients were diagnosed as GC between 1995 and 2006 at the Prince of Wales Hospital, and their formalin-fixed paraffin-embedded tissues were used in this study. Pathological diagnoses were performed by more than two pathologists. The CUHK Clinical Research Ethics Committee approved the usage of human samples and Reference No. is CREC 2018.343.

### RNA extraction and qRT-PCR

The related procedures have been indicated [13]. The primers for each gene are as follows: AMOT: F: 5′-ATT TTG CTC TGG ATG CTG CT-3′, R: 5′-TGG CCA TCA AGA TTT CTT CC-3′; AMOTL1: F: 5′-CGG GGA ACT TGT GAG CCT G-3′, R: 5′-CTG GGG AAA AGT AGG TGG AGT-3′; AMOTL2: F: 5′-GCT CGT TGA GTG AAC GGC T-3′, R: 5′-CAT GAG CTA GTA CAA CAT GAG GG-3′.

### Immunohistochemistry and immunocytochemistry staining

AMOTL1 (HPA001196, Sigma), YAP1 (ab52771, Abcam), CTGF antibody (sc-14939, Santa Cruz), Ki67 (550609, BD Pharmingen), and cleaved-Caspase 3 (#9664, CST) were commercially available. The immunohistochemistry and the scoring of the results were performed as previously described [13]. For immunocytochemistry studies, AGS was cultured on coverslips in a six-well plate. After removal of the medium and three times of PBS washing, the cells were fixed with 4% paraformaldehyde at room temperature for 15 min. Followed by three times of PBS washing, the cells were then permeated with 0.1% Triton X-100 at room temperature for 15 min, and another PBS washing three times. Room-temperature blocking was done with 2% BSA for 45 min; then cells were incubated with the primary antibody (1:200) at 4 °C overnight. Again, after one time of PBS washing, the cells were incubated with goat anti-mouse IgG secondary antibody (Alexa Fluor 594, 1:400, Thermo Fisher Scientific) in the dark at room temperature for 1 h. After washing, nuclei were stained with 4′,6-diamidino-2-phenylindole (DAPI, Thermo Fisher Scientific). Images were captured with a microscope (Carl Zeiss Axio Imager 2, Oberkochen, Germany).

### Plasmid construction

pcDNA3.1-Myc-His-AMOTL1 (Changsha Yingrun Biotechnology, China), p2xFlaghYAP1 (#17791, Addgene), pEGFP-C3-hYAP1 (#17843, Addgene), p2xFLAGhYAP1-WW mutant (#17792, Addgene), pL-CRISPR.EFS.GFP (#57818, Addgene), and pENTR1A (A10462, Invitrogen) were commercially available. The SFB-tagged destination (DEST) vector, together with the package plasmids pMD2G and pSPAX2, was a kind gift from Prof. Wenqi Wang (Department of Developmental and Cell Biology, University of California, Irvine, USA). HA-tagged Ubiquitin plasmid was generously given by Prof. Jun Yu (Institute of Digestive Disease, the Chinese University of Hong Kong). Based on pcDNA3.1-Myc-His-AMOTL1, the target fragment AMOTL1 (NM_130847) was generated using the following primers: F: 5′-AAC CAA TTC AGT CGA CGC CAC C AT GGA TCC CGG GCA GCA G-3′, R: 5′-AAG CTG GGT CTA GAT ATC TAA CCA TGT AAG AAA GCT TTC TTT ATC TAG CTT GG-3′. According to homologous recombination, between site SalI (R3138S, NEB) and EcorV (R3195S, NEB), the fragments were subsequently inserted into pENTR1A, from which the DEST vectors were created with the LR Clonase (#11791020, Invitrogen).

For the stable KO experiment, gRNAs and genomic cleavage detection (GCD) primers were designed on CHOPCHOP (http://chopchop.cbu.uib.no/) as follows: AMOTL1: gRNA-A1: GCC ATG ATC GCC TCA TGT GGA GG; GCD primers: F: GTG TGA ATG GGG TTG ATT GTC, R: CTG GTT ACC TTT CAC CGC AG; gRNA-A2: GAC CAT CTC GTG GAG CAT CCC GG, GCD primers: F: CAC CTG AGT ACC CCT TCA AGA C, R: CTA TTG AAT TTT GAA AAG CCG C; gRNA-A3: GTC AGC ACG CCA AGA ACC GCA GG; GCD primers: F: AAA CCT CAC TCA AGA AGA CCC A, R: CAT GTA GTA ACC ATG GCC CAC; gRNA-A4: CGA GGA ACT GCC CAC TTA CGA GG; GCD primers: F: CAA GAA CAC CAG GTG GAC AAT A, R: AGT TCG GGA CTT CTG ACT GGT. The gRNA oligos were annealed and combined into pL-CRISPR.EFS.GFP (BsmBI, R0580S, NEB) with Quick Ligase (M2200S, NEB).

### Expression profiling

AGS was treated with siScramble and siAMOTL1, respectively, followed by RNA extraction. The RNA samples were sent to Macrogene (Korea) for expression microarray analysis as indicated [12].

### Western blot analysis

The primary antibodies of TEAD1 (sc-376113), TEAD4 (sc-134071), and CTGF (L-20) (sc-14939) were from Santa Cruz (Dallas, TX, USA). YAP1 (ab52771) antibody and HA tag (ab18181) were achieved from Abcam (Cambridge, MA, USA). AMOTL1 (HPA001196) and Flag-tag (F3165) antibodies were obtained from Sigma-Aldrich (St. Louis, MO, USA). Other primary antibodies were from Cell Signaling (Danvers, MA, USA), including p21 (#2946), p27 (#2552), pRb (Ser807/811) (#9308), Erk (#9102), pErk (#9101), Myc (#2278), cyclin D1 (#2978), c-Myc (#9402), and GAPDH (#2118). Anti-Mouse IgG-HRP (Dako, Glostrup, Denmark, 00049039, 1:30,000) and anti-Rabbit IgG-HRP (Dako, 00028856, 1:10,000) were used for secondary antibodies. The related protocol was suggested before [13].

### Cell transfection and functional assays

siAMOT (SI00295386), siAMOTL1 (SI03156286), and siAMOTL2 (SI04195030) were commercially obtained from Qiagen (Valencia, CA). All transfection assays were performed using Lipofectamine™ 2000 Transfection Reagent (Invitrogen). The related procedures, including flow cytometry for cell-cycle distribution and apoptosis analysis, have been indicated previously [40]. For the CRISPR/Cas9-mediated knockout (KO) assay, 293TN cells were transfected with pL-CRISPR.EFS.GFP (gRNA sequence inserted), pSPAX2, and pMD2G. The medium was applied for BGC-823. Detection of the GFP signal in BGC-823 cells indicated a successful viral transfection. Both Western blot and DNA sequencing were used to verify KO efficiency.

### Drug-sensitivity tests

The drug-sensitivity tests were performed according to the cell viability rate, and were evaluated by the corresponding IC_50_ values. We treated cells with negative control, and siAMOTL1, respectively, with Cisplatin, 5-FU, and VP (Sigma-Aldrich, St. Louis, MO, USA), respectively. Controls were treated with an equal amount of vehicle DMSO (Sigma-Aldrich, St. Louis, MO, USA).

### Animal studies

Xenograft-formation assays were performed as the previous work [13]. Tumor sizes were measured every other day after 1 week. Tumor weights were acquired on day 21. All procedures were approved by the Department of Health, Hong Kong, and CUHK Animal Ethics Committee. The Reference No. is 17-492 in DH/SHS/8/2/1 Pt.4.

### Statistics

The statistical methods in this study for comparison and correlation were as previously described [13]. The student *t* test was used to compare the expression level of AMOT family in the TCGA cohort, as well as the functional differences between siRNA and control-treated cells. Survival was indicated by Cox regression. All statistical analyses were performed by SPSS software (Version 22.0, SPSS Inc.). A two-tailed *P* value of < 0.05 was considered statistically significant, and the *P* value < 0.001 was highly significant. Unsupervised clustering was performed through the R project.

## Supplementary information

Supplementary file
