# Supplementary material for: AMOTL1 enhances YAP1 stability and promotes YAP1-driven gastric oncogenesis
Source: Oncogene. 2020 Apr 20;39(22):4375–89. doi: 10.1038/s41388-020-1293-5 (PMC7253359; doi:10.1038/s41388-020-1293-5)
Supplement: Supplementary file 1 — Supplementary file [file 41388_2020_1293_MOESM1_ESM.pdf]

Figure S1

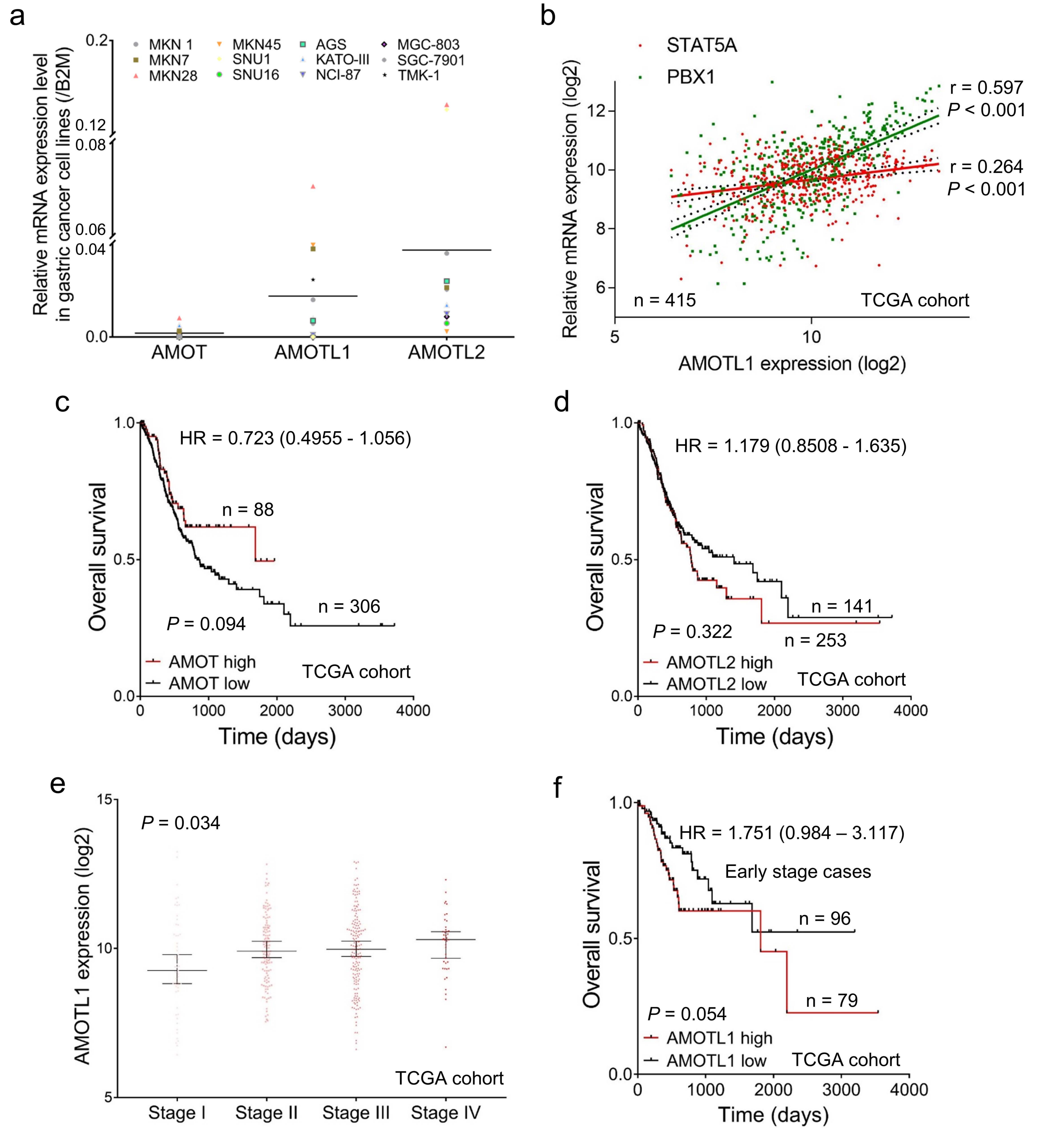

Figure S1 The expression pattern of AMOT family members in GC cell lines and primary cases. **a** Expression levels of AMOT, AMOTL1 and AMOTL2 in 12 GC cell lines. **b** Potential transcriptional factors of AMOTL1. **c** and **d** Overall survival rates regarding to AMOT and AMOTL2 expression in TCGA cohort. **e** AMOTL1 expression is increasing based upon more advanced clinical stage. **f** Overall survival rate based on AMOTL1 expression in early stage of GC cases.

Figure S2

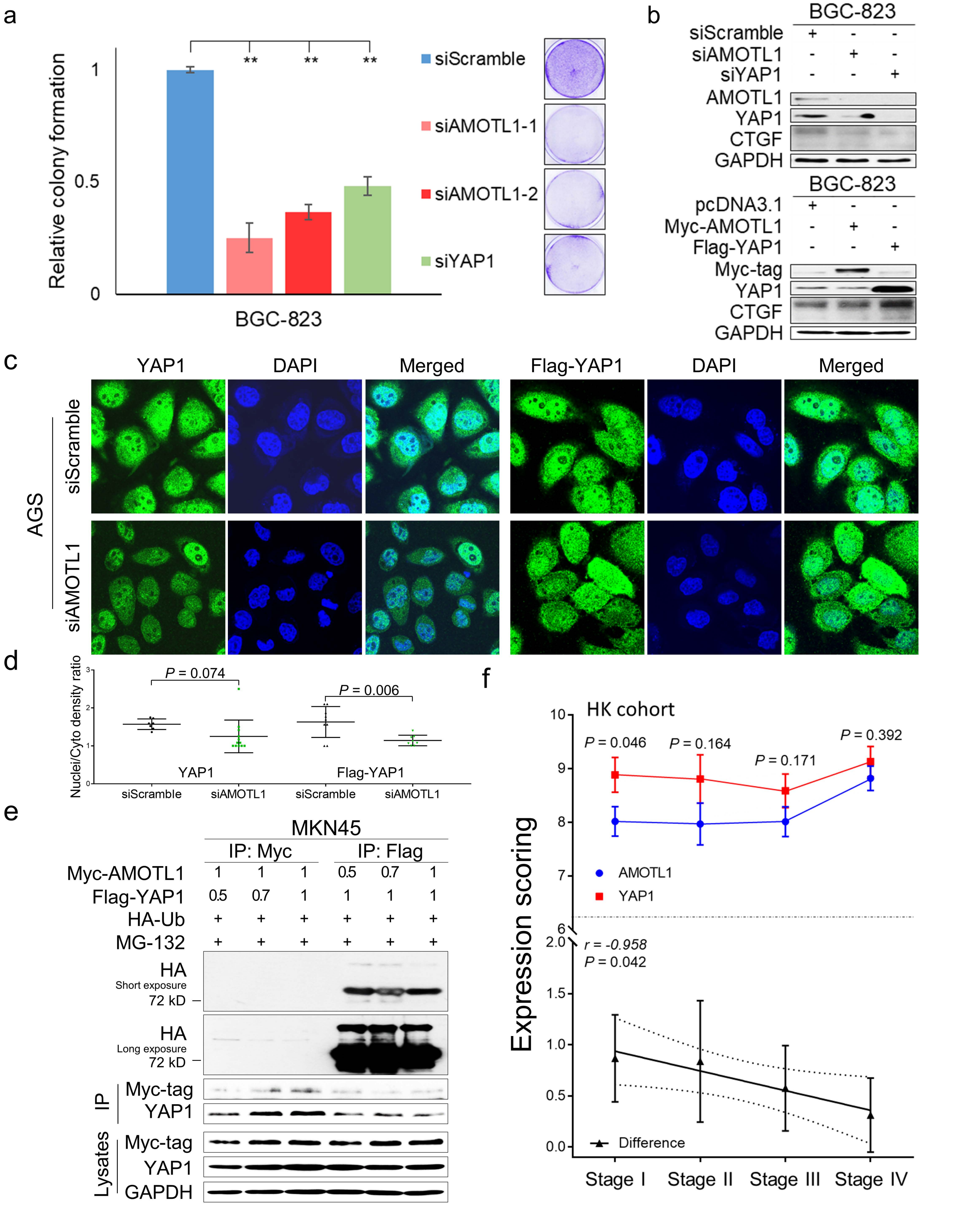

Figure S2 Interaction between AMOTL1 and YAP1 promotes their stabilization. **a** Either AMOTL1 KD or YAP1 KD in BGC-823 cells led to suppression of colony formation. **b** Either AMOTL1 or YAP1 regulates CTGF expression. **c** and **d** Both endogenous and exogenous YAP1 were isolated in the cytoplasm with siAMOTL1 transfectants. **e** AMOTL1-YAP1 binding prevented the ubiquitination of YAP1. Meanwhile, YAP1 also protected AMOTL1 from ubiquitin-mediated degradation. **f** The expressions of AMOTL1 and YAP1 demonstrate a consistent pattern in advanced stages of GC.

Supplementary Table S1. Correlation of AMOTL1 expression in GC with other clinicopathologic features in TCGA cohort (significant *P*-value in bold and Italic format). The case number and percentage counted were shown in the table.

		TCGA cohort (n = 321)				
		AMOTL1 high		AMOTL1 low		*P*-value
Sex	M	97	(48.50%)	103	(51.50%)	0.336
	F	52	(42.98%)	69	(57.02%)	
Age	≤60	59	(54.63%)	49	(45.37%)	***0.036***
	>60	90	(42.25%)	123	(57.75%)	
Type	Intestinal	81	(38.76%)	128	(61.24%)	***<0.001***
	Diffuse	68	(60.71%)	44	(39.29%)	
Stage	1	13	(30.95%)	29	(69.05%)	0.087
	2	47	(45.19%)	57	(54.81%)	
	3	69	(48.94%)	72	(51.06%)	
	4	20	(58.82%)	14	(41.18%)	
Stage (T)	1	0	(0.00%)	11	(100.00%)	***0.009***
	2	29	(42.03%)	40	(57.97%)	
	3	69	(48.25%)	74	(51.75%)	
	4	51	(52.04%)	47	(47.96%)	
Stage (N)	0	39	(39.00%)	61	(61.00%)	
	1	46	(48.94%)	48	(51.06%)	0.273
	2	27	(46.55%)	31	(53.45%)	
	3	37	(53.62%)	32	(46.38%)	
Stage (M)	0	138	(46.15%)	161	(53.85%)	0.727
	1	11	(50.00%)	11	(50.00%)	
Lymph Node	0	39	(39.00%)	61	(61.00%)	0.073
	1	110	(49.77%)	111	(50.23%)	

Supplementary Table S2. Correlation of AMOTL1 expression in GC with other clinicopathologic features in Hong Kong cohort (significant *P*-value in bold and Italic format). The case number and percentage counted were shown in the table.

		Hong Kong cohort (n = 273)				
		AMOTL1 high		AMOTL1 low		*(Spearman)* *P*-value
Sex	M	75	(41.21%)	107	(58.79%)	0.939
	F	37	(40.66%)	54	(59.34%)	
Age	≤60	49	(47.57%)	54	(52.43%)	0.525
	>60	63	(37.06%)	107	(62.94%)	
Type	Intestinal	57	(38.51%)	91	(61.49%)	***0.009***
	Diffuse	54	(43.55%)	70	(56.45%)	
Grade	1	3	(37.50%)	5	(62.50%)	***0.001***
	2	40	(39.22%)	62	(60.78%)	
	3	68	(41.98%)	94	(58.02%)	
Stage	1	31	(54.39%)	26	(45.61%)	***0.014***
	2	8	(25.00%)	24	(75.00%)	
	3	36	(41.86%)	50	(58.14%)	
	4	36	(37.11%)	61	(62.89%)	
Stage (T)	1	19	(52.78%)	17	(47.22%)	0.323
	2	31	(40.79%)	45	(59.21%)	
	3	59	(40.41%)	87	(59.59%)	
	4	2	(14.29%)	12	(85.71%)	
Stage (N)	0	32	(55.17%)	26	(44.83%)	0.063
	1	24	(34.78%)	45	(65.22%)	
	2	33	(39.76%)	50	(60.24%)	
	3	22	(35.48%)	40	(64.52%)	
Stage (M)	0	94	(41.05%)	135	(58.95%)	***0.005***
	1	17	(39.53%)	26	(60.47%)	
Lymph Node	0	32	(55.17%)	26	(44.83%)	0.227
	1	79	(36.92%)	135	(63.08%)	
H. Pylori	0	65	(53.72%)	56	(46.28%)	0.261
	1	41	(29.29%)	99	(70.71%)	

Supplementary Table S3. Relative expression levels of gene list on AGS cell line after siYAP1 treatment.

ProbeID	Gene_Symbol	Gene_ID	mRNA Accession	siYAP1 VS siControl
16730503	YAP1	10413	NM_001130145	-7.53308
17023646	CTGF	1490	NM_001901	-3.70106
16685036	ZMYM6NB	1.01E+08	NM_001195156	-3.49011
16815953	MIR4718	1.01E+08	NR_039869	-3.37951
16913617	MIR548O2	1.01E+08	NR_039605	-3.34425
16889627	SNORD70	692110	NR_003058	-3.12314
16724995	OR1S1	219959	NM_001004458	-2.87705
16697119	ARPC5	10092	NM_001270439	-2.75876
17005865	HIST1H2BM	8342	NM_003521	-2.71882
16902809	FAR2P2	1E+08	NR_046258	-2.65126
16696094	ANKRD36BP1	84832	NR_026844	-2.6362
16876251	KIR3DP1	548594	OTTHUMT00000478052	-2.62892
16728729	MIR4692	1.01E+08	NR_039841	-2.57742
16810011	MIR2116	1E+08	NR_031750	-2.56142
17107309	VGLL1	51442	NM_016267	-2.56087
16775701	LOC105370288	1.05E+08	XM_011508416	-2.5408
16788764	MIR544A	664613	NR_030257	-2.44526
16669374	HSD3B1	3283	NM_000862	-2.41777
16880667	MIR4433A	1.01E+08	NR_039632	-2.41686
16919862	MKRN7P	7686	NR_026640	-2.41526
16907572	GPR1	2825	NM_001098199	-2.39725
16989408	MIR4461	1.01E+08	NR_039666	-2.38801
17043698	LOC105375152	1.05E+08	XR_927032	-2.38727
16833706	MIR4727	1.01E+08	NR_039880	-2.34917
16865094	MIR519A1	574496	NR_030218	-2.3098
16938296	SGOL1-AS1	1.01E+08	NR_132785	-2.27666
16882655	LOC101928403	1.02E+08	XR_245043	-2.26775
16797447	IGHM	3507	BC011857	-2.26389
16670172	LOC100505824	1.01E+08	XR_171210	-2.25706
16685868	CITED4	163732	NM_133467	-2.21718
17086978	LOC101928014	1.02E+08	XR_242646	-2.19555
16986244	NSA2	10412	ENST00000610426	-2.18852
16797921	MKRN3	7681	ENST00000568945	-2.17374
17016748	HLA-F-AS1	285830	NR_026972	-2.16502

17068720	LOC100287846	1E+08	NR_037168	-2.09732
16763966	SNORA2A	677793	NR_002950	-2.09254
17111114	SPANXN5	494197	NM_001009616	-2.08756
16933027	DRICH1	51233	XM_011530207	-2.06462
16865018	MIR1323	1E+08	NR_031568	-2.05716
16927749	IGLV1-44	28823	OTTHUMT00000321110	-2.04816
16989367	DDX46	9879	ENST00000507053	-2.04661
16788622	SNORD114-4	767580	NR_003196	-2.04564
17005858	HIST1H2AI	8329	NM_003509	-2.04196
16892860	LOC105373953	1.05E+08	XR_924034	-2.03276
17069381	LOC101929628	1.02E+08	XR_928964	-2.03194
17000518	HSPA9	3313	AK023317	-2.03102
16788741	MIR654	724024	NR_030390	-2.01779
16734284	KRTAP5-2	440021	NM_001004325	-2.0051
16821033	NPIPB15	440348	NM_001306094	-2.00346
16660906	TMEM50A	23585	NM_014313	-2.00107
16798812	ARHGAP11B	89839	OTTHUMT00000430733	2.00233
16694188	ASH1L	55870	NM_018489	2.003458
16861016	GPR42	2866	OTTHUMT00000347518	2.007361
16864819	ZNF480	147657	NM_001297624	2.012475
16851353	MIB1	57534	NM_020774	2.013283
16862866	ZNF221	7638	NM_001297588	2.025035
16896196	XDH	7498	NM_000379	2.028303
17013507	SAMD5	389432	NM_001030060	2.034171
16883760	SLC9A4	389015	NM_001011552	2.037258
16806279	HERC2	8924	NM_004667	2.039487
16890126	PIKFYVE	200576	NM_001178000	2.043306
16846745	SPAG9	9043	NM_001130527	2.047982
16744991	MPZL3	196264	NM_001286152	2.056374
16907979	ABCA12	26154	NM_015657	2.057522
16789530	LINC00638	196872	NR_024396	2.058104
16881242	OR7E91P	79315	NR_002185	2.064185
16731490	LOC105369502	1.05E+08	XR_913542	2.065902
16909695	LOC100286922	1E+08	NR_037694	2.070053
17061881	DOCK4	9732	NM_014705	2.075573
16734650	LOC101927708	1.02E+08	NR_126335	2.075789
16998332	LOC105379096	1.05E+08	XR_948598	2.07882
16791734	HEATR5A	25938	NM_015473	2.079495

16801695	C2CD4A	145741	NM_207322	2.080188
16968521	MIR4451	1.01E+08	NR_039656	2.080678
17048246	AKAP9	10142	NM_005751	2.081997
16847352	LOC653653	653653	NR_027408	2.082133
17052530	MGAM2	93432	NM_001293626	2.084653
17042915	TFAMP1	260341	NR_001288	2.0884
16798553	MIR4509-1	1.01E+08	NR_039732	2.090222
16805940	MIR4509-1	1.01E+08	NR_039732	2.090222
16806388	MIR4509-1	1.01E+08	NR_039732	2.090222
16764160	KMT2D	8085	NM_003482	2.098054
16991008	MIR378A	494327	NR_029870	2.100085
16924143	BAGE2	85319	OTTHUMT00000157418	2.103585
17092875	IFNE	338376	NM_176891	2.112009
16709268	ACSL5	51703	NM_016234	2.116655
16971791	LOC100505784	1.01E+08	XR_939399	2.135755
16779855	MYCBP2	23077	NM_015057	2.141359
16979225	PRSS12	8492	NM_003619	2.147839
16952118	TRANK1	9881	NM_014831	2.168744
17005573	HIST1H2BD	3017	NM_021063	2.172979
16872313	ZNF780B	163131	NM_001005851	2.174011
16917004	GPCPD1	56261	NM_019593	2.181336
17013283	UTRN	7402	NM_007124	2.186991
17083988	MTAP	4507	ENST00000427788	2.187831
16875952	VN1R1	57191	NM_020633	2.19321
16912898	MIR4755	1.01E+08	NR_039911	2.199751
16872029	LGALS4	3960	NM_006149	2.203796
16998162	MCTP1	79772	NM_001002796	2.204519
16818015	SNORA30	677813	NR_002966	2.210226
17051194	LINC01000	402483	NR_024368	2.218264
17052552	MGAM2	93432	NM_001293626	2.223023
17117521	LOC102723539	1.03E+08	XR_428894	2.223385
16704182	CSGALNACT2	55454	NM_018590	2.225154
16806561	LOC102725021	1.03E+08	XM_011508479	2.229356
17013512	SAMD5	389432	XM_011535819	2.236291
16664579	MIR4421	1.01E+08	NR_039617	2.23796
16861553	ZNF570	148268	NM_001300993	2.241119
16698972	LOC105372902	1.05E+08	XR_920738	2.247202
16864917	VN1R2	317701	NM_173856	2.249853

16984689	ITGA2	3673	NM_002203	2.259748
16797929	LOC105370733	1.05E+08	XR_916102	2.282198
16681862	PRAMEF10	343071	BC112208	2.315646
16810177	VPS13C	54832	NM_001018088	2.332408
16753543	MIR548C	693129	NR_030347	2.336672
16982083	SLED1	643036	NR_003542	2.348896
16828918	LOC105371389	1.05E+08	XR_917567	2.369629
16818927	CHD9	80205	NM_001308319	2.37699
16808268	CATSPER2	117155	NM_001282309	2.385793
17063507	KDM7A	80853	NM_030647	2.435183
16979917	SLC7A11	23657	NM_014331	2.547136
17094496	MIR1299	1E+08	NR_031629	2.56166
17012388	LOC105377991	1.05E+08	XR_942964	2.593115
17075060	MIR548V	1E+08	NR_036103	2.641862
17117684	NPIPB11	728888	XM_011546005	2.724656
16779790	CTAGE11P	647288	NR_027466	2.751316
16730215	PIWIL4	143689	NM_152431	2.752873
16900286	LINC00342	150759	AK057596	2.787487
16851230	ANKRD20A5P	440482	NR_040113	2.915083
17092640	RNU6-14P	1.01E+08	ENST00000384630	3.105346
16758610	MIR3908	1.01E+08	NR_037470	3.291011
17090564	SNORD62A	26786	NR_002914	3.341366
17090566	SNORD62A	26786	NR_002914	3.341366
16803754	CEMIP	57214	NM_001293298	3.342561
17012711	SNORA33	594839	NR_002436	3.728941
17006743	SNORA38	677820	NR_002971	4.371444
17083433	IL33	90865	NM_001199640	6.605803

Supplementary Table S4. Univariate and multivariate Cox regression analysis of the association between clinicopathologic characteristics and disease specific survival in GC patients (significant *P*-value in bold and Italic format).

	TCGA cohort (n = 321)	
	Univariate analysis	Multivariate analysis
Sex	0.452	-
Age	0.069	***0.003***
Type	0.086	0.664
Stage	***<0.001***	0.101
Stage (T)	***0.001***	0.396
Stage (N)	***0.001***	0.779
Stage (M)	***0.008***	0.189
Lymph Node	***0.001***	0.200
AMOTL1	***0.016***	-
YAP1	0.705	-
TEAD4	0.337	-
CTGF	***0.008***	-
CYR61	***0.016***	0.704
AMOTL1+CTGF	***0.019***	***0.008***

Supplementary Table S5. Univariate and multivariate Cox regression analysis of the association between clinicopathologic characteristics and disease specific survival in GC patients (significant *P*-value in bold and Italic format).

	Hong Kong cohort (n = 273)	
	Univariate analysis	Multivariate analysis
Sex	0.286	-
Age	***0.037***	***0.002***
Type	***<0.001***	0.803
Grade	***0.006***	0.172
Stage	***<0.001***	***<0.001***
Lymph node metastasis	0.136	-
AMOTL1	***<0.001***	-
Nuclear YAP1	***<0.001***	-
CTGF	***<0.001***	-
AMOTL1+CTGF+nuclear YAP1	***0.001***	***<0.001***
